# Refusals to Provide Anesthesia for Abortion Care: Reconsidering Conscientious Objection Claims Among Anesthesiologists

**DOI:** 10.1213/ANE.0000000000008057

**Published:** 2026-04-28

**Authors:** Jacob M. Nieb, Katie Watson, Alyssa Burgart

**Affiliations:** From the 1 Department of Anesthesiology; 2 Medical Humanities & Bioethics Graduate Program, Departments of Medical Education, Medical Social Sciences, and Obstetrics and Gynecology, Northwestern University Feinberg School of Medicine, Chicago, Illinois; 3 Department of Anesthesiology, Perioperative & Pain Management, Stanford Center for Biomedical Ethics, Stanford University School of Medicine, Palo Alto, California.

An obstetrician-gynecologist (OB-Gyn) practicing in a state that protects abortion care was performing an abortion in a hospital procedure room separate from the main operating room when the young, healthy patient experienced anaphylaxis while under anesthesia. The anesthesiologist called for additional help, but the on-call anesthesiologist refused to come because the procedure was an abortion. The OB-Gyn was upset by what they believed was patient abandonment and a violation of the principle that conscientious objection does not override a duty to care in emergencies. However, when they raised this issue with leaders of the hospital’s anesthesiology department, their concern “was shrugged off. ‘Eh, this person doesn’t do these cases, so what can you do?’”

Anesthesiologists may exercise a conscientious objection to providing care for patients undergoing abortions.^1^ However, confidential communication to one author (KW) has alerted us to concerning cases in which a conscientious objection standard is incorrectly applied, such as anesthesiologists refusing to provide care in life-threatening situations (see Table). A nationwide survey of 169 OB-Gyn residency program directors found that 69% faced opposition to providing abortion care in their practice settings, attributing 30% to anesthesiologist opposition.^2^ Conscientious objection is an established safeguard for an individual’s personal integrity. However, the way some anesthesiologists may apply it in abortion-related care raises urgent ethical and practical concerns. Siddiqui et al correctly state that anesthesiologists “should not be asked to forsake moral beliefs as a condition of practice.”^3^ Simultaneously, patients should not be asked to forsake standard-of-care medical treatment based on which anesthesiologist happens to be on call. According to the American College of Obstetricians and Gynecologists (ACOG), “Safe, legal abortion is a necessary component of women’s health.”^4^ Safe procedural and induction abortion includes pain management needs. Therefore, anesthesiologists must develop and operationalize conscientious objection protocols that eliminate potential negative impacts on patients, including delays in access to routine abortion care, and threats to patient life and health in emergency abortion care.

**Table.** Each of These Cases Involve an Unfounded Refusal to Provide Care in Which the Anesthesiologist Has a Professional Obligation to Provide Care Regardless of Their Personal Feelings About the Case

Scenario	Problem	Solution
Overbroad procedure-based objections	The anesthesiologist has a core objection to abortion because he does not want to participate in “killing a fetus.” For this reason, he refuses to provide anesthesia for D&Cs. A patient presents with absent fetal heart tones and needs a D&C for management of her missed abortion, which he refuses.	This professional needs to work through objection in advance and commit to maximizing their contribution in cases that do not cross their moral lines/violate their integrity. If he has not done so, his supervisors need to be prepared to swiftly and clearly explain that he won’t be excused from service because this isn’t a conscientious objection.
Moral discomfort	The anesthesiologist is uncomfortable with abortion and would not personally have one, and she wants to object due to this discomfort.	This professional needs to carefully review her discomfort and recognize that, although she does not personally like abortion care, this dislike does not undermine her moral integrity, and therefore she provides anesthesia for the procedure. As described above, ideally this review is done before the case arises. If she does object, a replacement may have to be found because the conversation her supervisors will need to have with her is more detailed.
Emergency situations (obstetric)	The anesthesiologist refuses to provide anesthesia for emergency D&E for a patient with a septic abortion in the setting of PPROM despite no other available anesthesiologist to provide care.	The anesthesiologist has a valid conscientious objection, which is ethically and legally overridden by the patient’s need for emergency care. Unless an alternate clinician can substitute without adding any moment of delay to the case, the clinician must provide care despite their conscientious objection.
Emergency situations (non-obstetric)	The anesthesiologist is caring for a patient undergoing a D&E. The patient becomes acutely hypotensive and hypoxic with concern for anaphylaxis. He calls for help, and another anesthesiologist refuses to assist because the procedure is a D&E.	The second anesthesiologist has a valid conscientious objection, but this objection is ethically and legally overridden by the patient’s need for emergency care.
Legal concerns	The anesthesiologist is practicing in a state that bans all abortions except in cases of threat to the life of the mother. The hospital procedures for determining if a patient meets these criteria have been followed for a patient who presents for a D&C. The anesthesiologist on duty is not morally opposed to abortion, but declines to provide anesthesia for a D&C because she does not want her name on the patient’s record saying “I’m not going to jail for this.”	The anesthesiologist’s concerns for their own civil or criminal liability should be analyzed under a conflict of interests framework. They do not a constitute conscientious objection. A clinician’s independent analysis of legal risk that runs contrary to hospital or departmental procedures for this analysis and that has no sound, independent legal basis is not an ethical basis for refusing to provide care.

To support anesthesiologists’ ethical contribution to abortion care and their ethical use of conscientious objection,^5^ in this paper we define conscientious objection and discuss refusals to provide care contextualized in the practice of anesthesiology. We discuss the anesthesiologist’s role in abortion care as it relates to patient safety and advocacy. Lastly, we articulate a model of conscientious objection that centers on patients without denigrating physicians and examines failures in patient care through a quality improvement lens.

This article focuses on individual conscience and conscientious objection. Questions of institutional conscience (for example, a Catholic hospital that refuses to allow most abortions on-site) and conscientious provision are beyond its scope. We also take no position on the question of whether anesthesia provision during abortion is “direct care” that is within the scope of conscientious objection policies. Instead, because it is common to assume anesthesiologists can ethically object to participating in abortion care, our analysis presumes this position.

## THE ANESTHESIOLOGIST’S ROLE IN ABORTION CARE

The ethics of abortion care is a subject of debate in the United States (US), so it’s no surprise that physicians also have a range of views on the topic. This alone should inspire thoughtful development of conscientious objection policies. However, recent dramatic changes in US state abortion laws and an increase in patient travel have added new complexity for some anesthesiology practices. The US Supreme Court’s landmark 2022 decision in *Dobbs v Jackson Women’s Health Organization* reversed *Roe v Wade* (1973), and the loss of a federal constitutional right to abortion has allowed individual states to restrict or permit access to abortion, leading to a variety of conflicting laws across the country. In this article, states that protect abortion access until at least 18 weeks of gestation are referred to as “access states” (currently 30), and states with total abortion bans (currently 15) or bans after 6 weeks (currently 3) or 12 weeks of gestation (currently 2) are referred to as “ban states.”

The changes in state laws have led to a significant increase in patients traveling to seek abortion care. This has resulted in some hospitals in access states performing more abortion procedures,^6,7^ and the need to raise money and arrange for travel can lead patients to present at later gestational ages.^8^ For example, in a confidential communication with author KW, an OB-Gyn in an access state estimates that before *Roe v Wade* was reversed in 2022 their hospital did 1-4 induction abortions per year for in-state residents, and now it does 1 to 8 *per week*, mostly for out-of-state residents. Finally, in 2024, the nationwide number of procedural and medication abortions provided by clinicians (a number that excludes self-managed abortions) was higher than before *Roe* was reversed.^9^ In ban states, the closure of clinics shifts any abortion procedures permitted by life-and-health exceptions to hospitals. Both factors indicate an increase in the total number of hospital-based abortions with anesthesiologist support, adding another reason to revisit this topic.^10^

Anesthesiology professionals have important roles to play during procedural abortions: dilation and curettage (D&C), which typically occurs up to 13 weeks of gestation, and dilation and evacuation (D&E), which typically occurs at 14 weeks of gestation and later. D&C and D&E procedures involve mechanical cervical dilation and removal of products of conception. Induction abortion is another method used at 14 weeks and later after a natural or an induced intrauterine fetal demise, and anesthesiologists are frequently involved in the provision of analgesia for these patients. The Clinical Practice Guidelines of the National Abortion Federation (NAF, the professional organization of clinicians working at independent abortion clinics) explicitly call for the provision of anesthesia for procedural and induction abortions.^11^ These guidelines outline the need for an expert in sedation, such as an anesthesiologist, who can rescue patients from deeper planes of sedation than intended. They also list the necessary safety equipment and medications in the event of an intraoperative emergency, a role with which anesthesiologists are comfortable.^11^ The World Health Organization recommends the option for analgesia during abortion, and the Society of Family Planning (SFP) also released clinical guidelines that reiterate the need for anesthesia clinicians in abortion procedures, noting that 79% of first-trimester procedural abortion clinicians prefer that an anesthesiology professional provide moderate sedation, deep sedation, or general anesthesia over local anesthesia alone.^12–14^ The safety of deep sedation and general anesthesia in first- and second-trimester abortions has been affirmed in large reviews, and anesthetic considerations for abortion are further detailed in Ozery et al.^15,16^

Emergency procedures are in a different category, and the requirement to assist is a matter of role morality (the role of the physician) versus personal morality. Obtaining anesthesia for many other routine and urgent procedures is usually simple, but in abortion care, an anesthesiologist’s conscientious objection can lead to delays in obtaining indicated care or to patients receiving unnecessarily painful procedures without the recommended anesthesia. This negatively impacts patients, other clinicians, and the healthcare system more broadly.^17^ Abortion is a time-sensitive procedure–any delay in care can increase risks and costs, and given that the timeframe for legal abortion is geographically variable, delays can also put patients beyond their legal window to exercise reproductive autonomy.

## CONSCIENTIOUS OBJECTION AND THE DUTY TO CARE FOR PATIENTS

“Conscience” refers to the deeply held, foundational beliefs of a physician as a person.^18^ Occasionally, a physician’s conscience may conflict with accepted standards of medical care, which can lead to a fractured sense of self and moral injury. Conscientious objection occurs when a physician refuses to provide standard medical care on the basis of conscience.^19^ ACOG defines conscience more broadly than most statutory protection, and therefore we use its description in this article: “An appeal to conscience would express a sentiment such as ‘If I were to do ‘x,’ I could not live with myself/I would hate myself/I wouldn’t be able to sleep at night.’ According to this definition, not to act in accordance with one’s conscience is to betray oneself—to risk personal wholeness or identity.” Perhaps equally importantly, ACOG clarifies what conscientious objection is *not*: “distaste for certain procedures, discriminatory attitudes, or other self-interested motives. Providers who decide not to perform abortions primarily because they find the procedure unpleasant or because they fear criticism from those in society who advocate against it do not have a genuine claim of conscience.”^19^

In the US, enforcement of federal laws protecting physician conscience is delegated to the US Department of Health and Human Services, and enforcement procedures are codified in the Final Rule, “Safeguarding the Rights of Conscience as Protected by Federal Statutes.”^20^ Most recently updated in 2025, the Final Rule states that healthcare workers can conscientiously object to providing or assisting in the performance of care, but it no longer defines the scope of what “assisting in the performance of care” entails, leaving that determination to the healthcare clinician or institution.^20,21^

The law is one-sided: it protects a physician’s right to conscientiously object to participation in abortion care, but it does not prescribe the course of action that should be taken to ensure patient care in the event of objection.^20^ Ethics codes pick up this baton, and both the ACOG Ethics Committee Opinion and the American Medical Association (AMA) Code of Medical Ethics place the onus on the physician who objects. As the AMA states, “Physicians should refer a patient to another physician or institution to provide treatment the physician declines to offer.”^18^ For anesthesiologists, this duty to “refer” requires the objector to identify a willing colleague who can provide timely care, and the “referral” should often be made to that willing anesthesiology colleague rather than the patient.

Conscience may ethically be used as a shield to protect a physician’s personal integrity. It is unethical to use it as a club to attempt to restrict the options or behaviors of patients.^22^ This distinction explains why the American Society of Anesthesiologists (ASA) recognizes the anesthesiologist’s obligation to the patient even if the anesthesiologist objects to a patient’s decisions. As stated in the ASA Ethical Guidelines (I.2): “Anesthesiologists respect the right of every patient to self-determination. [...] Anesthesiologists should not use their medical skills to restrain or coerce patients who have adequate decision-making capacity.”^23^

By the time an anesthesiologist is called on to provide anesthesia for an abortion, the patient has already made a medical decision that is affirmed by their OB-Gyn. A refusal to provide anesthesia therefore undermines the patient’s autonomy, and an anesthesiologist who refuses to provide anesthesia should find a timely replacement to avoid any disruption of care. Because the need to find a new anesthesiologist may result in delays and costs, preventing the need to find a last-minute replacement is an important consideration in this discussion.

## PREVENTATIVE ETHICS

Investing in “preventative ethics” can eliminate the need for referral, or it can enable a seamless referral. Anesthesia departments, group practices, and training programs should consider requiring and supporting the following practices. For an anesthesia clinician who objects to assisting in abortion care, the first step in a preventative approach is tailoring one’s objections as narrowly as possible to maximize one’s clinical contribution. For example, some anesthesiologists might refuse to assist in abortion care only after a certain gestational age; others might refuse in a pregnancy that could result in a healthy birth at any gestational age but assist in incomplete or missed abortions with present fetal heart tones where there is no possibility of sustained life outside the womb. Next, the anesthesiology clinician should disclose their specific objections to their superiors and colleagues, identify a contingency plan to allow for conscientious objection without impacting a patient’s medical care, and confirm they understand their ethical obligation to assist in emergencies regardless of conscience. For some anesthesiology clinicians, “preventative ethics” could even mean joining a practice where the probability of providing anesthesia for abortions is extremely low–for example, specializing in cardiac anesthesia or chronic pain, or working in a large enough practice where an individual’s objection is counterbalanced by the number of clinicians who do not object.

## EMERGENCY SITUATIONS

Although the Final Rule, the AMA, ACOG, and the ASA recognize the right to conscientious objection, it is always subject to an important ethical limitation: emergency scenarios. The Final Rule states that conscientious objection provisions must still be consistent with the Emergency Medical Treatment & Labor Act, and that physicians must provide emergency care regardless of objections.^20^ The AMA states, and ACOG and the ASA affirm, that physicians must “provide care in emergencies.”^18,23^ The case described at the opening of this article, in which a patient receiving an abortion experienced an anesthetic emergency and the on-call anesthesiologist refused to assist, is a clear misapplication of conscience, because the principle that conscientious objection does not override a duty to care in emergencies remains in procedural abortions that were initially expected to be routine. This case highlights the need for further education that emergency care is ethically compulsory regardless of an underlying objection to the procedure being performed.

The definition of “emergency” can be complex since in some abortion cases medical emergencies unfold over time and in unpredictable patterns, and this concept deserves further discussion aimed at reaching consensus at every institution. There is also a different category we think of as “structural emergencies” that anesthesiologists must consider carefully, which is when delays may diminish or entirely eliminate a patient’s ability to receive care. For example, a patient nearing a state’s gestational age limit who cannot afford to travel to a more permissive jurisdiction, or a patient who has traveled across state lines and can only stay in town for a set amount of time, do not meet the definition of medical emergency but they are in the midst of structural emergencies that lead to life- and health-altering consequences.

## OBJECTION FOR REASONS OTHER THAN CONSCIENCE

Sometimes an anesthesiologist’s objections to participating in abortion care are related to reasons other than conscientious objection. These include overbroad procedure-based objections, moral discomfort, and legal concerns. These objections are often incorrectly labeled as “conscientious,” and it is important to distinguish them because they are not legitimate grounds for refusing to provide care. Examples of each are further detailed in the Table.

## OVERBROAD PROCEDURE-BASED OBJECTIONS

Some clinicians mistakenly approach conscientious objection as a binary: an anesthesiologist either participates in abortion care or they do not. We do not treat other procedures this way, and similarly anesthesiologists should not refuse to participate in all D&C and D&E procedures, because the indications and medical details are relevant for conscientious objection. For example, if the indication for D&C is absent fetal heart tones, then an anesthesiologist’s refusal to participate based on conscientious objection is nonsensical–the treatment (D&C) does not cause fetal demise, and therefore the basis for a claim of conscientious objection is absent. Conscientious objection should also not be used when treating complications from abortions. For example, if a patient who had a medication abortion elsewhere presents with excess bleeding, the goal of removing the remaining pregnancy tissue is not ending her pregnancy. The abortion has already occurred, and the goal is to preserve the patient’s life and health by appropriately managing a complication.

Details surrounding the indication for abortion have been shown to matter to anesthesiologists.^24^ In a semi-structured qualitative interview study of anesthesia clinicians in the Southeastern United States, Reeves et al elicited clinicians’ thoughts on hypothetical abortion cases.^24^ One of the cases involved a patient with preterm, premature rupture of membranes (PPROM) at 21 weeks’ gestation. With no other information, 6 of the 15 anesthesia clinicians objected to providing anesthesia for this abortion. After clarifying the poor prognosis for the fetus and risks to the patient with continuing the pregnancy, two of the six changed their decisions and agreed they would provide care.^24^ This study demonstrates that context and facts lead to anesthesia clinicians’ informed decision-making on matters of conscience. It is this informed decision-making, rather than a binary approach of procedure-based conscientious objection, that we should be striving for.

The simplicity of a personal policy of “nothing that has anything to do with abortion” may be an appealing way to avoid potentially uncomfortable conversations or moments, but it is also unethical. Many anesthesiologists have not received sufficient training in the nuances of abortion care, and providing the information, encouragement to engage with it, and space to discuss the relationships between medical facts and personal beliefs is ideally a role that will be played by group leadership. Anesthesiologists owe it to their patients and their colleagues to thoughtfully define the boundaries of their objections, and to expand their practice as widely as possible.

## MORAL DISCOMFORT

Philosopher Mark Wicclair differentiates conscientious objection from objections based on peripheral moral beliefs, which influence a person’s attitudes, but are not central to their core beliefs. He argues that providing care that runs counter to peripheral moral beliefs may lead to feelings of moral discomfort but it does not rise to the level of fracturing one’s sense of self.^21^ Some anesthesiologists may be uncomfortable with abortion care but not to the level required of conscientious objection—for example, they may be concerned about other people’s negative perceptions of abortion care or they may feel conflicted about the provision of abortion. This is not the same as having a core opposition to abortion care.

## LEGAL CONCERNS

Refusal to provide anesthesia due to a fear of legal prosecution is well documented.^25,26^ Although it may sometimes be understandable, it is not a conscientious objection–it is a conflict of interest, in which an anesthesiologist prioritizes a small chance of harm to themselves over a high chance of harm to their patient. OB-Gyn Jennifer Reeves has authored several studies that examine anesthesia clinicians’ attitudes toward abortion care.^24,25,27^ In a study examining the impact of the 22-week abortion ban Georgia had at the time, she found that “fear of criminality” was a prominent theme among all the anesthesiologists interviewed.^25^

The negative impact this fear has on patients was highlighted in a case in which an OB-Gyn practicing in a state with an abortion ban and a “medical emergency” exception shared with one author (KW). A patient presented with progressive dyspnea and was ultimately diagnosed with diffusely metastatic cancer with pleural effusions. She was given an initial prognosis of 6 months, and immediate chemotherapy was recommended. She had been attempting to conceive and was found to be five weeks pregnant when she received her cancer diagnosis, but because the chemo regimen would be harmful to her embryo, she decided to end her pregnancy and begin treatment as soon as possible. The institution’s requirement that two physicians agree on what constitutes a “medical emergency” under state law was completed. The maternal fetal medicine and complex family planning physicians decided the abortion should proceed in the OR due to the patient’s medical complexity and tenuous respiratory status, and the patient was evaluated in the pre-operative clinic by an anesthesiologist. However, a different anesthesiologist was assigned to her procedure on the day the case was scheduled and refused to provide care, citing the new legislation and concern for criminalization as their reason for “conscientious objection.” As a result, the patient did not receive the depth of sedation (and accompanying amnestic effects) that the physicians doing the procedure wanted, and she did not have an anesthesiologist present should her respiratory status have worsened or should she have experienced an airway emergency. Instead, she had her manual vacuum aspiration in a triage bed with a paracervical block and minimal sedation without the presence of a clinician with anesthesia expertise, resulting in the potential for adverse patient harm due to her medical comorbidities.

Anesthesiologists must follow the laws in their respective states, which can be challenging with rapidly changing and ambiguous laws. For this reason, it is imperative that institutions create a framework to evaluate and document the legality of abortion in particular cases (for example, via on-call ethics and/or legal consultation), thus removing the onus of legal interpretation from the individual anesthesiologist and relieving concerns about prosecution. Hospitals need legal support and interpretations that prioritize patient safety and enhance the provision of ethical care.

## WHEN CONSCIENTIOUS OBJECTION WORKS

Having examined scenarios that do not reflect conscientious objection, we present a hypothetical case of an effectively and ethically implemented conscientious objection.

On reviewing her cases the night before, an anesthesiologist sees that she is assigned to an OR with a gynecologist performing D&E procedures. She sees that the first case is indicated for absent fetal heart tones, and the subsequent cases are procedural abortions for otherwise uncomplicated pregnancies. She has a deeply held moral belief against abortion. She knows the first case might make her sad or uncomfortable but does not object to it because the fetus is nonviable. She objects to the remaining cases because although anesthesia is not the direct care that ends embryonic or fetal life, anesthesia facilitates the procedure that does, and therefore she feels that providing anesthesia in these cases would contradict her deeply held beliefs. She contacts her colleague assigned to the room next to her about trading assignments, as well as the OR coordinator to see if they have an alternative case assignment she can take in exchange for her assigned room. Between her colleague and the coordinator, a change in assignments is made in the evening and both rooms start on time the next morning. The patient doesn’t know anyone objected to their care, thereby reducing stigmatization and delay, and the physician performing the abortion does not either, reducing staff friction. The assignment switch also means her objection neither lightens her workload nor increases the workload of others.

This example works for multiple reasons: the cases are scheduled, there are clinicians readily available to trade assignments, the anesthesiologist has carefully thought through her personal values and understands the ethical boundaries of conscientious objection, and the physicians involved conduct themselves in a way that prioritizes patient autonomy.

For the add-on surgical abortion or for practices that do not use a substantial number of anesthesiologists who will participate in abortion care, this seamless transfer of care may be difficult, but anesthesiologists in this circumstance must find new ways to make it possible. We turn now to examine arguments for the application of conscientious objection and mitigating pitfalls for unscheduled cases.

## WORKFORCE RECIPROCITY IN ANESTHESIA

One insufficiently discussed issue is the strain conscientious objection places on the healthcare system, and how the objector can and should be required to mitigate that strain through an expectation of reciprocal work. Conscientious objection in medicine is modeled on conscientious objection in wartime, and that model does not excuse service altogether. For example, objectors during World War II were still required to assist in noncombatant roles such as medics.^28^ In a review of the impact of conscientious objection provisions on both patients and clinicians, de Londras et al found 15 studies examining workload impacts that found a disproportionate increase in workload for clinicians who provide abortion care.^17^ This must be changed. Those who object should still expect to work—by trading assignments, exchanging calls, providing breaks or lunches, or helping with preoperative evaluations. As stated in the ASA Ethical Principles (III.1), “Anesthesiologists should promote a cooperative and respectful relationship with their professional colleagues that facilitates quality medical care for patients. This responsibility respects the efforts and duties of other care clinicians.”^23^ Put simply, anesthesiologists and trainees who opt out of abortion care need to increase their contributions elsewhere in compensation for the patient care their colleagues do on their behalf. Practically speaking, work hours and break times should not significantly differ among these clinicians, nor should call requirements.

**Figure.**
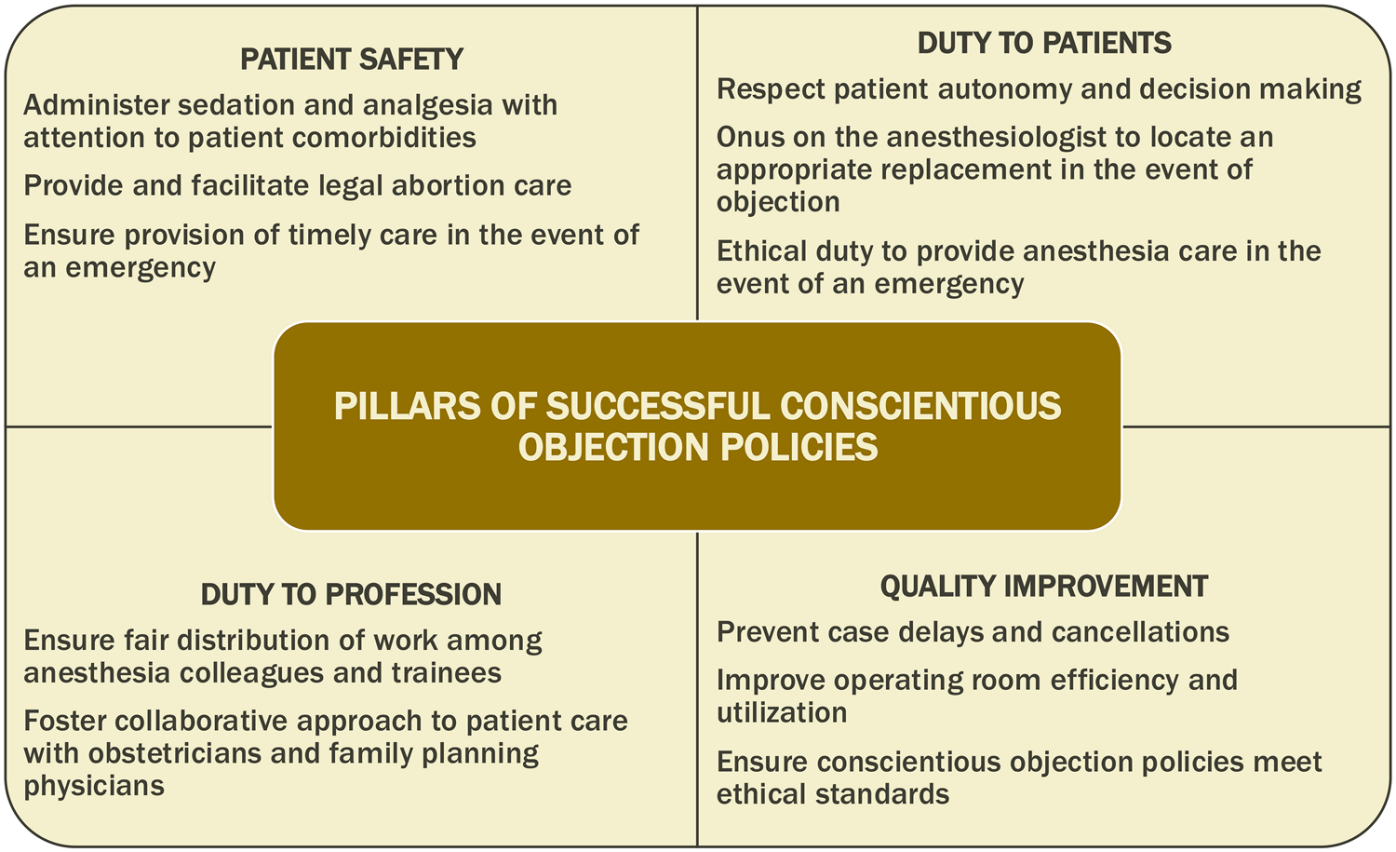
Considerations for conscientious objection policies and summary of best practices.

One author (JN) experienced a second structural defect of one hospital’s conscientious objection policy in his anesthesiology residency that goes beyond the issue of workload and impacts training. He was willing to provide care during abortions, and the extra coverage he provided for conscientious objectors at times led him to miss opportunities for rare cases simply because abortions were also scheduled. Like overwork, the unintended “penalty” of missing training opportunities because one provides abortion care that allows colleagues to maintain their conscience must be recognized and eliminated. In addition to striving for balance in work hours, training programs should strive to ensure a trainee’s experience does not significantly differ due to covering for conscientious objection.

## SCHEDULING AND PATIENT SAFETY

Abortion care is not something done exclusively in daytime hours, and staffing models must accommodate this uncertainty. As previously stated, conscientious objection is limited in the event of an emergency, and thus care should not be delayed for emergency abortions. However, patient safety concerns are relevant in “routine” abortion care as well. Delays can result in abortions later in pregnancy, exposing patients to additional physiologic changes and increased risk of morbidity.^17^ In addition, the emotional and psychological harm that could occur from delayed care, or from an abortion performed without adequate (or any) anesthesia, could be significant. All these patient harms should be considered by anesthesiologists when defining their conscientious objection to abortion care.

## POLICY OPPORTUNITIES

We recommend that anesthesiology practices consider their current policies regarding conscientious objection. Pillars of a successful policy include: attending to patient safety, respecting patient autonomy, articulating obligations to provide care during emergencies, and encouraging each anesthesiologist to thoughtfully identify and communicate the parameters of their potential objections. Additionally, policies provide an opportunity to agree on how colleagues will address workforce reciprocity, foster collaborative patient care, and attend to quality concerns (Figure).

## FUTURE DIRECTIONS

Conscientious objection is legally protected in the US and has a strong ethical underpinning in our medical practice. However, an anesthesiologist’s objection should never interfere with emergency care nor abandon patients presenting with complications from abortion. Anesthesiologist’s conscientious objection should not unduly burden patients presenting for routine care and should not unduly burden their colleagues within anesthesiology and across the drapes.

To continue caring for the field’s vulnerable patients and fostering relationships with our obstetrical colleagues, anesthesia practices and training programs need to discuss abortion openly and more frequently, and they should establish conscientious objection policies and facilitate values clarification sessions to encourage thoughtful application of their policies. These policies should be implemented and evaluated in the same manner as quality improvement initiatives. Practices should track how many abortions are delayed or canceled due to conscientious objection (or misapplied objections that are permitted to stand), how long these cases are delayed, and any adverse outcomes experienced by patients. These delays are entirely preventable with appropriate staffing models, call structures, and when the onus of finding an anesthesiologist who will provide care for the patient is placed on the objecting anesthesiologist. At a time when the legal status of abortion is geographically variable and changing, we as anesthesiologists should deeply consider our moral beliefs regarding abortion, decide if these feelings rise to the level of conscientious objection, and stand up for all patients to ensure they have access to care.

## DISCLOSURES

**Conflicts of Interest:** None. **Funding:** None. **This manuscript was handled by:** Adam J. Milam, MD, PhD.

## ACKNOWLEDGMENTS

Every confidential communication described in this article is shared with the clinician’s permission, and we thank them for sharing these anonymously..
